# Insights into distorted lamellar phases with small-angle scattering and machine learning

**DOI:** 10.1107/S1600576725000317

**Published:** 2025-02-11

**Authors:** Chi-Huan Tung, Lijie Ding, Guan-Rong Huang, Lionel Porcar, Yuya Shinohara, Bobby G. Sumpter, Changwoo Do, Wei-Ren Chen

**Affiliations:** a Neutron Scattering Division Oak Ridge National Laboratory,Oak Ridge TN37831 USA; b Department of Engineering and System Science National Tsing Hua University,Hsinchu300044 Taiwan; c Physics Division, National Center for Theoretical Sciences, Taipei10617, Taiwan; d Institut Laue–Langevin, B.P. 156, F-38042GrenobleCedex 9, France; e Materials Science and Technology Division Oak Ridge National Laboratory,Oak Ridge TN37831 USA; f Center for Nanophase Materials Sciences Oak Ridge National Laboratory,Oak Ridge TN37831 USA; NSRRC, Taiwan

**Keywords:** small-angle scattering, machine learning, distorted lamellar phases, regression analysis, Kolmogorov–Arnold networks

## Abstract

Lamellar phases are essential in various soft matter systems, with topological defects significantly influencing their mechanical properties. In this report, we present a machine-learning approach for quantitatively analyzing the structure and dynamics of distorted lamellar phases using scattering techniques. By leveraging the mathematical framework of Kolmogorov–Arnold networks, we demonstrate that the conformations of these distorted phases – expressed as superpositions of complex waves – can be reconstructed from small-angle scattering intensities. Through the contour analysis of wave field phase singularities, we obtain the statistics of the spatial distribution of topological defects. Furthermore, we establish that the temporal evolution of these defects can be derived from the time-dependent traveling wave field, informed by the dispersion relation of spectral components. This method opens new avenues for investigating the dynamics of distorted lamellar phases using various dynamic scattering techniques such as neutron spin echo and X-ray photon correlation spectroscopy. These findings enhance our microscopic understanding of how defects influence the physical properties of lamellar materials, with implications for both equilibrium and non-equilibrium states in general lamellar systems.

## Abstract 2

This study employs Kolmogorov–Arnold networks to analyze neutron and X-ray scattering from distorted lamellar phases within a wave field representation, identifying topological defects and structural transitions. It offers insights into defect structures and their impact on material properties.

## 1. Introduction

Lamellar phases, characterized by a regular, layered arrangement of molecules, are commonly found in a variety of soft materials and biological systems, including self-assembled block copolymers, surfactants, liquid crystals and membrane systems. These phases are crucial for the material properties and behaviors of complex systems, making their structural characterization a key area of research. Small-angle scattering (SAS) techniques, particularly those using neutrons and X-rays, have been applied extensively to study lamellar structures (Prévost *et al.*, 2017 ▸; Porte, 2002 ▸). Ideal lamellar models (Nallet *et al.*, 1993 ▸; Zhang *et al.*, 1994 ▸; Lemmich *et al.*, 1996 ▸; Pabst *et al.*, 2000 ▸, 2003 ▸; Bouglet & Ligoure, 1999 ▸; Mihailescu *et al.*, 2002 ▸; Castro-Roman *et al.*, 2005 ▸) assume perfect lamellar ordering (Vonk, 1978 ▸) and are effective in systems where the layers remain uninterrupted.

However, the above assumption about the perfect lamellar ordering is questionable in many cases. For example, electrical conductivity measurements (Photinos *et al.*, 1981 ▸; Boden *et al.*, 1981 ▸; Photinos & Saupe, 1986 ▸, 1991 ▸; Boden & Jolley, 1992 ▸), NMR spectroscopy (Callaghan & Soderman, 1983 ▸; Davis, 1983 ▸; Chidichimo *et al.*, 1987 ▸, 1988 ▸; Ukleja *et al.*, 1991 ▸; Coppola *et al.*, 1995 ▸, 2003 ▸; Jóhannesson *et al.*, 1996 ▸; Hubbard *et al.*, 2005 ▸; Eriksson *et al.*, 2015 ▸) and transmission electron microscopy (TEM) imaging (Kléman *et al.*, 1977 ▸; Meyer *et al.*, 1978 ▸; Bourdon *et al.*, 1982 ▸; Costello *et al.*, 1984 ▸; Allain & Kléman, 1985 ▸, 1987 ▸; Allain, 1986 ▸; Kléman, 1989 ▸; Strey *et al.*, 1990 ▸; Blanc *et al.*, 2005 ▸; Moreau *et al.*, 2006 ▸; Zhang *et al.*, 2012 ▸) show that topological defects frequently introduce distortions and discontinuities within the lamellar layers. These defects can compromise structural integrity, alter flow behavior and affect light transmission, highlighting the limitations of the ideal lamellar model in accurately describing the behavior of distorted phases.

In ideal lamellar models, the density profile along the direction normal to the interface is represented by square waves, and the measured scattering intensity is modeled as the Fourier transform of the autocorrelation of this profile. However, in the presence of perforations between neighboring layers or crumpled surfaces, the density profile no longer follows this form. As a result, the ideal models are insufficient for describing the behavior of distorted lamellar phases, which deviate from the ideal lamellar ordering due to these topological disruptions.

Intriguingly, these topological disruptions can cause distorted lamellar phases to exhibit conformational features similar to sponge structures, another important class of lyotropic phases. SAS techniques have been used to quantify sponge phase conformation through Berk’s inversion algorithm (Berk, 1987 ▸, 1991 ▸), which models density fluctuations as a superposition of plane waves with randomly distributed wave vectors. In contrast, the ideal lamellar model represents density fluctuations as plane waves with wave vectors aligned along the normal direction of the interfaces. This similarity suggests a promising approach to model distorted lamellar structures as intermediate forms between the ideal lamellar and sponge phases, where the key variable to control is the wave vector distribution.

SAS techniques using neutrons (Kékicheff *et al.*, 1984 ▸; Hendrikx *et al.*, 1984 ▸, 1987 ▸; Kékicheff & Cabane, 1988 ▸; Leaver & Holmes, 1993 ▸; Holmes *et al.*, 1993 ▸) and X-rays (Paz *et al.*, 1984 ▸; Holmes & Charvolin, 1984 ▸; Boden *et al.*, 1986 ▸; Holmes *et al.*, 1987 ▸, 1988 ▸; Kékicheff, 1989 ▸; Kékicheff & Tiddy, 1989 ▸; Boden *et al.*, 1990 ▸; Funari *et al.*, 1992 ▸, 1994 ▸; Quest *et al.*, 1994 ▸; Fairhurst *et al.*, 1997 ▸; Dhez *et al.*, 2000 ▸; Minewaki *et al.*, 2001 ▸; Orädd *et al.*, 2001 ▸; Castelletto *et al.*, 2002 ▸; Yamashita *et al.*, 2004 ▸; Baciu *et al.*, 2007 ▸; Angelov *et al.*, 2009 ▸; Meklesh & Kékicheff, 2021 ▸) have proven effective for investigating lamellar systems. These methods are sensitive to disruptions in smectic ordering caused by topological defects, revealing characteristic signatures in the diffuse scattering profiles (Spinozzi *et al.*, 2010 ▸; Spinozzi & Amaral, 2016 ▸). However, accurately quantifying the impact of these defects remains challenging (Hamley, 2022 ▸). Moreover, extracting key statistical descriptors – such as defect classification, volume density and spatial heterogeneity – from static two-point correlation functions is difficult due to the nonlinear relationship between topological properties and the scattering function.

To address these challenges, a machine-learning approach is proposed, wherein density fluctuations in lamellar phases are modeled as a superposition of plane waves with controlled wave vector distributions. A comprehensive library of two-point correlation functions for lamellar phases is constructed to train a generative Kolmogorov–Arnold network (KAN), which facilitates the inversion of real-space conformations from experimental scattering patterns. This approach enables the extraction of statistical and dynamical properties of distorted lamellar phases.

The feasibility of this deep-learning strategy is demonstrated both computationally and experimentally. Topological defects manifest as disruptions in the regular stacking of layers, with spatial continuity in complex wave fields perturbed by phase singularities. A contour integral approach is applied to identify these defects by locating phase singularities in wave representations inverted from SAS data. This paper introduces a novel methodology for statistically characterizing the spatial arrangement and dynamical properties of topological defects in lamellar phases. It offers new insights into the molecular-level behavior of these complex materials and discusses the potential of machine learning to enhance scattering data analysis, laying the groundwork for future advancements in soft matter scattering research.

## 2. Wave field representation of distorted lamellar structures

The wave field approach for modeling distorted lamellar phases originates from Turing (1952 ▸), who used plane wave superposition to explain biological pattern formation. Turing showed that small fluctuations in morphogen concentrations could form stable patterns through reaction–diffusion processes, breaking spatial symmetry and generating periodic structures without pre-existing templates. The plane waves capture the spatial modes of these fluctuations, leading to complex patterns defined by characteristic wavelengths.

Turing’s principle also underpins the understanding of soft matter systems. In 1987, Berk applied this framework to model density fluctuations in lyotropic bicontinuous microemulsions (Berk, 1987 ▸, 1991 ▸; Zemb, 2002 ▸), introducing the leveled wave approach. In this model, random plane waves in an isotropic medium mirror the dynamics of Turing’s morphogens, with the density fluctuations represented as a superposition of plane waves:
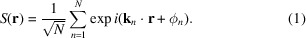
The real part of *S*(**r**) represents local density fluctuations, with random wave vectors 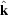 and uniformly distributed phase shifts 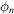. To define phase interfaces, a threshold parameter α is introduced, where the surface is defined by
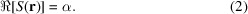
Regions where 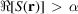 and 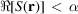 are distinguished by different scattering length densities. 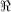 stands for the real part. The scattering length density distribution is defined using the Heaviside function *H*,
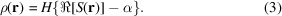
Finally, the Fourier transform of the autocorrelation of ρ(**r**) gives the coherent scattering intensity *I*(*Q*), linking the wave-based structural model to experimental scattering data. We represent the SLD as binarized values to highlight the mesoscopic distributions of the lamellae and water. For a more precise depiction of the detailed SLD distribution, the Heaviside function *H* in equation (3) can be substituted as needed to account for the chemical characteristics of the system.

To extend the plane wave superposition framework to distorted lamellar phases, we revise the distribution of wave vectors 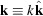 in equation (1) to account for the inherent anisotropy of the lamellar phases, where the normal vectors of the interfaces deviate from a random orientation. The anisotropic nature of density fluctuations necessitates the imposition of a constraint on the orientational order of **k**. In spherical coordinates, this distribution can be expressed as
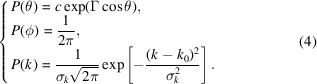
Here, θ is the polar angle, ϕ is the azimuthal angle and *k* is the magnitude of the wave vector.

Unlike the isotropic distribution assumed in equation (1), the polar component *P*(θ) in lamellar phases is characterized by an exponential distribution, as proposed by Fisher (1953 ▸). This function incorporates an order parameter Γ that quantifies the degree of directional anisotropy and a normalization constant *c* to account for smectic ordering. The angular distribution *P*(ϕ) remains uniform over the range of 2π, reflecting the absence of a preferred in-plane direction for density fluctuations at the interface. Following Berk’s original formulation (Berk, 1987 ▸, 1991 ▸), the radial distribution *P*(*k*) is described by a normal distribution with mean *k*_0_ and standard deviation σ_*k*_, capturing the dispersion of the wave vector magnitude.

**Figure 1:**
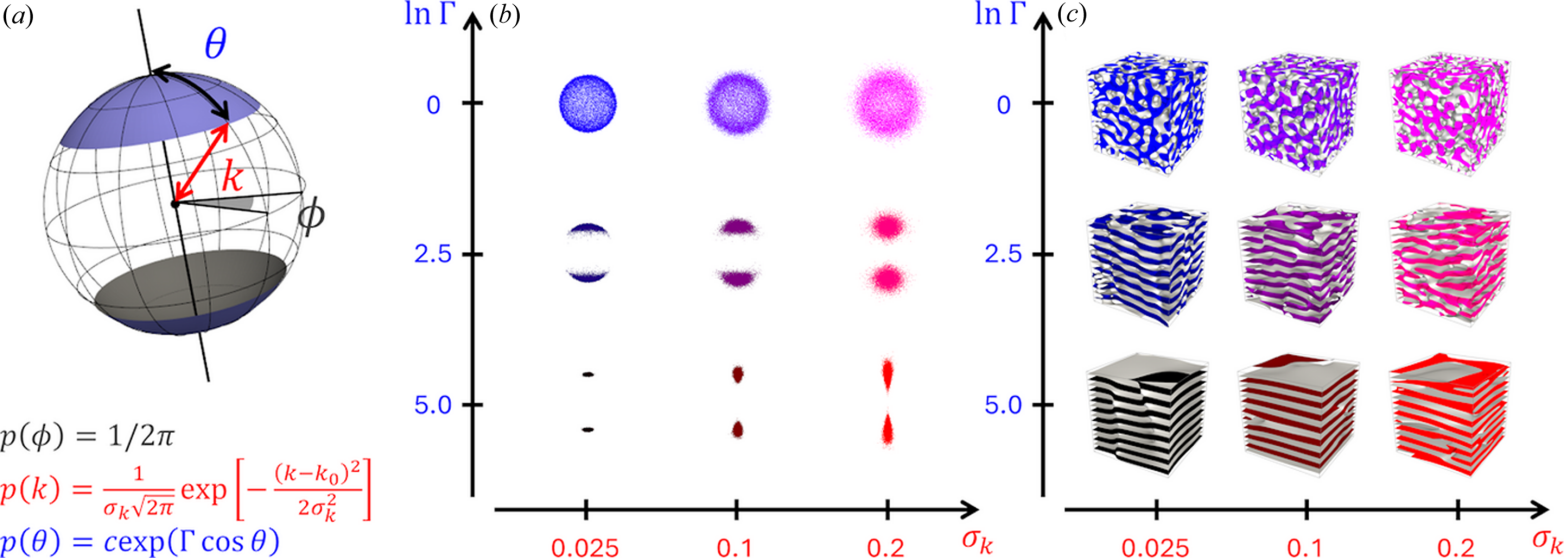
(*a*) Schematic of the anisotropic wave vector distribution defined in spherical coordinates. (*b*) Dependence of the wave vector distribution on the orientational order parameter in the polar angle direction (Γ) and the radial dispersion parameter (σ_*k*_). (*c*) Corresponding three-dimensional structures in real space, which are statistically equivalent to the wave vector distributions shown in (*b*), as obtained through ensemble averaging with α = 0.

Within this descriptive framework, each system is characterized by three primary parameters: σ_*k*_, Γand α. We define the characteristic length scale 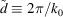 and assign the random phase ϕ_*n*_ following the uniform distribution in *U*(0, 2π). Fig. 1 ▸ demonstrates our generalized leveled wave (GLW) approach for 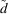 equal to 1/8 of the cell size, which generates real-space conformations of lamellar phases by modulating these parameters.

Fig. 1 ▸(*a*) illustrates the orientation regulation of the vector 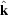 in spherical coordinates.

Fig. 1 ▸(*b*) shows the relationship between the anisotropic wave vector distribution and the parameters σ_*k*_ and Γ. For a fixed σ_*k*_, increasing Γ enhances polarization within the distribution of 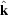, aligning the vectors more closely with the lamellar normal. Conversely, increasing σ_*k*_ while holding Γ constant results in a more diffuse vector distribution.

Understanding the influence of σ_*k*_, Γ and α on wave vector distributions enables us to interpret variations in the real-space conformations of distorted lamellar structures:

(i) The parameter σ_*k*_ governs the radial dispersion of *k*, quantifying the variability in inter-layer spacing among distorted lamellae.

(ii) The parameter Γ controls the angular dispersion in the polar direction, providing a measure of the degree of layer crumpling.

(iii) The clipping level α represents the ratio between the thickness of the amphiphilic molecular layer and that of the aqueous layer.

Fig. 1 ▸(*c*) displays three-dimensional interfacial conformations. The left column demonstrates that increasing Γ enhances the anisotropic order within the structure, while the bottom row illustrates that larger values of σ_*k*_ lead to greater variability in inter-plane distances.

**Figure 2:**
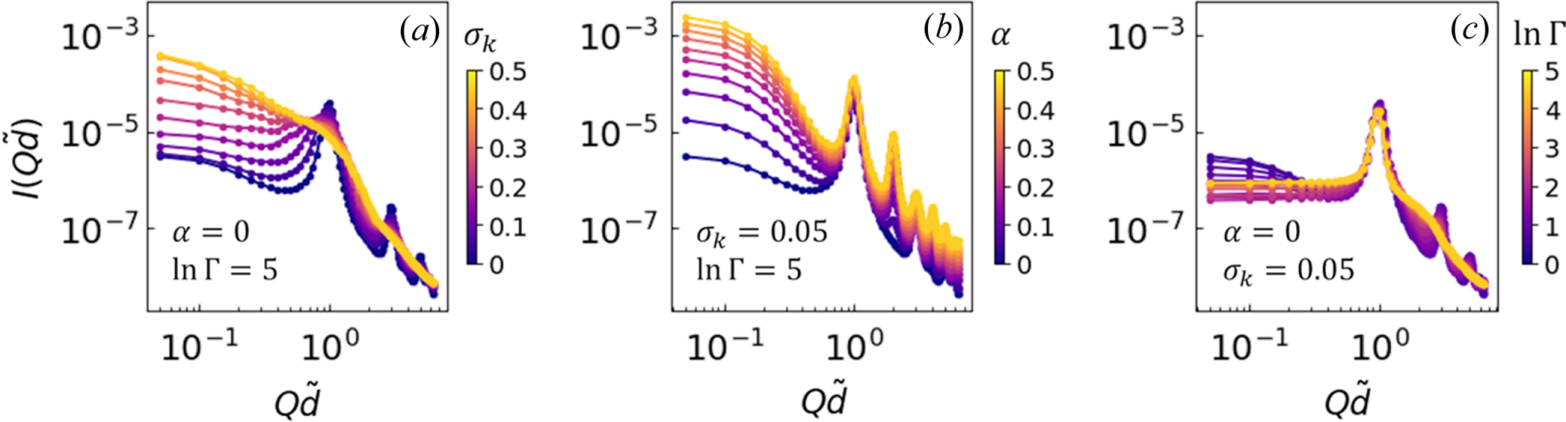
Dependence of SAS intensities on parameters σ_*k*_, Γ and α, shown as 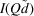 with the average inter-plane distance 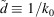: (*a*) With Γ = 128 and α = 0, increasing σ_*k*_ from 0 to 0.5 progressively broadens the three correlation peaks in 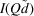 until they disappear, similar to the effect of increasing the *d* variation in the ideal lamellar model (Nallet *et al.*, 1993 ▸). (*b*) With Γ = 128 and σ_*k*_ = 0.05, increasing α from 0 to 0.5 reduces the intensity of even-numbered peaks, while odd-numbered peaks remain largely unaffected. This trend parallels findings from contrast variation SANS experiments (Doe *et al.*, 2009 ▸) and resembles the influence of δ in the ideal lamellar model (Nallet *et al.*, 1993 ▸). (*c*) With σ_*k*_ = 0.05 and α = 0, increasing ln Γ from 0 to 5 smears the higher-order peaks, similar to the effects observed with increasing σ_*k*_, though the primary peak remains unchanged. Unlike in the ideal model, variations in Γ effectively capture lamellar phase distortions.

The dependence of SAS intensities on the parameters σ_*k*_, Γand α is illustrated in Fig. 2 ▸. The coherent intensity is presented as 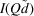, where 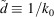 represents the average inter-plane distance in lamellar structures. To compute 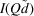, we first evaluate ρ(**r**) in a simulation cell of size 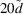, divided into a 512 × 512 × 512 grid. A fast Fourier transform is then applied to obtain the scattering amplitude *F*(*Q_x_*, *Q_y_*, *Q_z_*). Finally, *I*(*Q_x_*, *Q_y_*, *Q_z_*) is calculated as 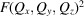, followed by radial averaging to obtain 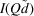.

As shown in Fig. 2 ▸(*a*), with Γ fixed at 128 and α = 0, an increase in σ_*k*_ from 0 to 0.5 results in a progressive broadening of the three correlation peaks in 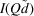, until they eventually vanish. This trend closely mirrors the behavior observed when increasing the variation in *d* within the ideal lamellar model (Nallet *et al.*, 1993 ▸). This similarity is anticipated, as σ_*k*_ in our GLW model controls the radial dispersion of *k*, and a higher σ_*k*_ naturally induces greater variability in the inter-layer spacing.

Fig. 2 ▸(*b*) demonstrates that, with Γ held at 128 and σ_*k*_ set to 0.05, an increase in α from 0 to 0.5 causes a gradual reduction in the intensity of the even-numbered correlation peaks, such as the second and fourth, until they eventually disappear, while the odd-numbered peaks remain largely unaffected. This distinctive trend aligns with findings from contrast variation small-angle neutron scattering (SANS) experiments on lamellar phases (Doe *et al.*, 2009 ▸). The observed behavior parallels the effect of modifying the parameter δ in the ideal lamellar model (Nallet *et al.*, 1993 ▸), which influences the phase relationship between neighboring layers.

In Fig. 2 ▸(*c*), with σ_*k*_ fixed at 0.05 and α at 0, increasing ln Γ from 0 to 5 leads to a smearing of the higher-order correlation peaks, similar to the effect observed with an increase in σ_*k*_. However, the intensity of the primary correlation peak remains unaffected. The evolution of 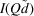 with changes in Γ, which quantifies the disruption of lamellar order, cannot be reproduced by adjusting either *d* or δ within the ideal lamellar model (Nallet *et al.*, 1993 ▸). This finding highlights the significance of our approach in accurately capturing the conformation of distorted lamellar phases.

## 3. Spectral inversion via Kolmogorov–Arnold networks

The primary question we address is whether it is possible to derive an analytical expression for the SAS intensity in terms of the three parameters, σ_*k*_, Γ and α, to invert the real-space conformation of distorted lamellar phases from experimentally measured *I*(*Q*). Specifically, we aim to determine if such an expression can be formulated in a manner analogous to the scattering functions for ideal lamellar phases (Nallet *et al.*, 1993 ▸) and sponge phases (Berk, 1991 ▸). However, due to the anisotropy in the wave vector distribution, this inversion problem presents a higher degree of complexity than is manageable by straightforward analytical approximation. The resulting problem is nonlinear and incorporates additional layers of complexity, making an exact analytical solution challenging to achieve.

To address the limitations of deriving the scattering function analytically for spectral regression analysis, we have developed several machine-learning approaches. These include Gaussian process regression (Chang *et al.*, 2022 ▸; Tung *et al.*, 2022 ▸; Ding *et al.*, 2024*c* ▸,*b* ▸,*a* ▸), variational autoencoders (VAEs) (Tung *et al.*, 2023 ▸) and convolutional neural networks (CNNs) (Tung *et al.*, 2024*b* ▸,*c* ▸), which have been applied to the spectral inversion analysis of various soft matter systems with complex structures that are not easily described by analytical models.

Although these machine-learning models demonstrate satisfactory performance on controlled test data, a significant limitation arises when applying them to real-world scattering data. The primary issue concerns the output *I*(*Q*). For example, within the CNN architecture (Tung *et al.*, 2024*b* ▸,*c* ▸), the output is generated as a one-dimensional array of *I*(*Q*) values with a fixed number of *Q* points, stemming from the fixed-size output imposed by the convolutional layers in the encoder. Unfortunately, this approach conflicts with real scattering experiments, where *I*(*Q*) is measured at variable *Q* points. As a result, to align the CNN’s fixed output grid with the experimental data’s variable *Q* points, we must rely on interpolation and extrapolation, which introduce inaccuracies in the inverted structural parameters and thus compromise the precision of the analysis. This limitation is a critical issue for handling real-world data, as interpolation and extrapolation introduce distortions in the relationship between the predicted and actual structural parameters, leading to significant sources of error.

To address these challenges, we introduce KAN (Liu *et al.*, 2024 ▸) as a flexible generative model that produces a continuous function for *I*(*Q*). This approach overcomes the limitations of fixed-output models, providing adaptability to real-world scattering data. Leveraging the Kolmogorov–Arnold representation theorem (Arnold, 1956 ▸; Kolmogorov, 1957 ▸), the KAN framework represents any multivariate continuous function as a finite sum of univariate functions of input variables. With the multivariate function 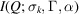 now directly represented by simpler univariate components without needing discrete convolution operations, KAN eliminates the requirement for a fixed output grid. This makes it particularly well suited for predicting scattering intensities *I*(*Q*) across varying *Q* points, thereby aligning naturally with experimental data.

The Kolmogorov–Arnold theorem (Kolmogorov, 1957 ▸) states that any multivariate continuous function can be represented as a superposition of simpler univariate continuous functions. This enables the decomposition of complex functions into more manageable components,
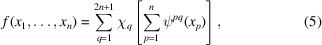
where 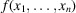 represents a multivariate continuous function, and 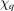 and 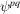 are univariate functions. In the context of the spectral inversion problem for distorted lamellar phases, each *x_p_* corresponds to conformational parameters such as σ_*k*_, Γ and α, along with the sampled wave vector points *Q*. Here, 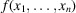 represents the experimentally measured scattering intensity 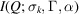.

In this study, both 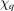 and 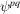 are represented using spline functions (Bartels *et al.*, 1987 ▸). The number of basis functions required for each is selected according to the resolution of the parameters σ_*k*_, Γ, α and *Q* within their respective ranges. The training process aims to determine the optimal coefficients for each spline basis function, using a training set of 8000 *I*(*Q*) values generated from the relevant ranges of σ_*k*_, Γ, α and *Q*.

**Figure 3:**
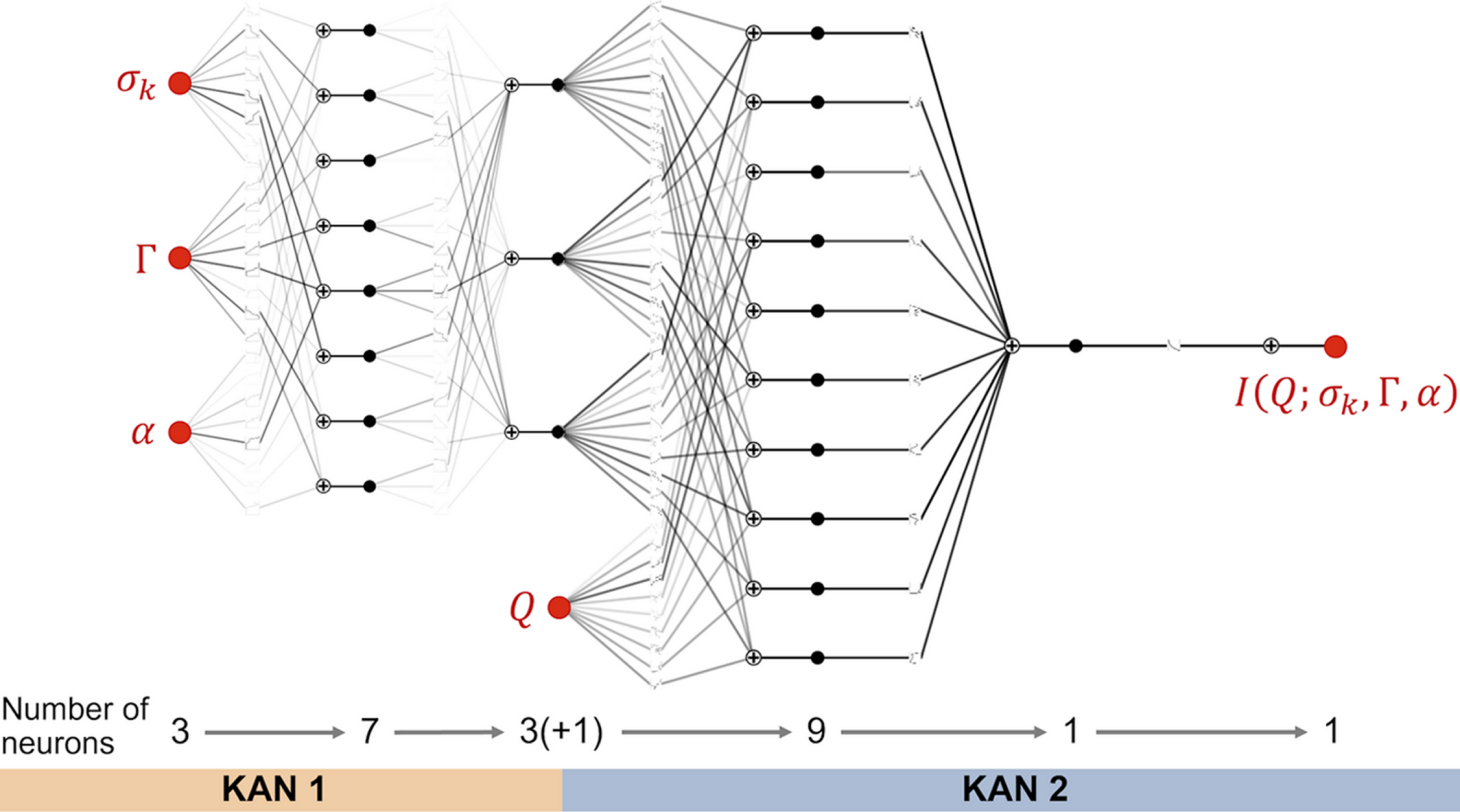
The KAN architecture models the relationship between scattering parameters σ_k_, Γ, α and the resulting scattering intensity *I*(*Q*) as a function of the wave vector *Q*. KAN 1 maps the scattering parameters into a higher-dimensional space using three layers (3, 7 and 3 neurons) to capture nonlinear dependencies. KAN 2 integrates *Q* with the KAN 1 outputs, using layers with 9, 1 and 1 neuron to predict 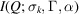, aligning with experimental data to reveal lamellar phase structures.

Building on equation (5), a two-module KAN architecture, shown in Fig. 3 ▸, is proposed to model the relationship between the structural parameters σ_*k*_, Γ, α and the scattering intensity *I*(*Q*) as a function of *Q*. This network comprises two sections, KAN 1 and KAN 2, each optimized with a distinct number of neurons to efficiently map the input structural parameters to the output intensity. The design is inspired by our previous unsupervised VAE study, which verified that a smooth mapping between *I*(*Q*) and its lower-dimensional representations is achievable, aligning well with KAN’s intended purpose. In this setup, KAN 2 represents the connection between *I*(*Q*) and its lower-dimensional representations, while KAN 1 links the structural parameters to quantities that can be decoded to generate *I*(*Q*).

In forward propagation, the key parameters σ_*k*_, Γ and α are first processed by KAN 1, which consists of layers with widths [3, 7, 3]. The output from KAN 1 yields three latent variables, which are then combined with *Q* to form a four-dimensional input for KAN 2, structured with layer widths [4, 9, 1, 1], producing the final output of the scattering intensity 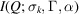. This functional relationship can be directly compared with experimental data, enabling a detailed analysis of lamellar phase structures through their scattering profiles.

The layered structure of this KAN model is designed to capture both the anisotropic order (through Γ) and the radial distribution (through σ_*k*_), while also considering the thickness ratio represented by α. This architecture provides a robust generative model that effectively maps complex relationships within the scattering data.

**Figure 4:**
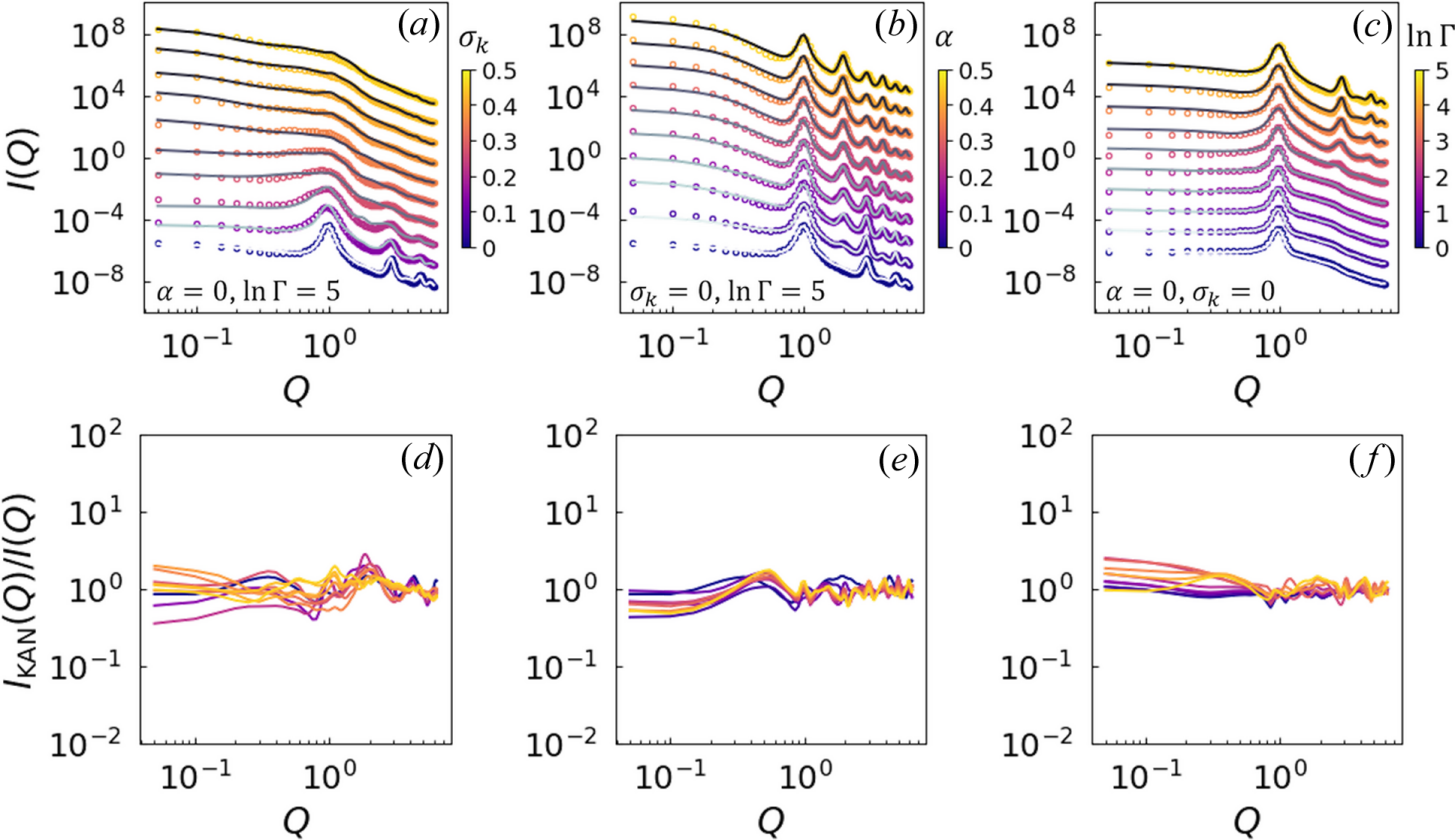
*I*(*Q*) generated using a separate set of parameters, (*a*) σ_*k*_, (*b*) α and (*c*) Γ, which were not included in the training dataset, to evaluate the accuracy of the KAN-based inversion algorithm. Panels (*d*)–(*f*) display the ratio between KAN-produced curves and the test set. The comparison reveals excellent quantitative agreement between the *I*(*Q*) values obtained from GLW (colored open symbols) and those generated by KAN (solid curves) across the examined parameter ranges. This agreement underscores the numerical precision and robustness of the KAN-based regression method.

Using a separate set of parameters σ_*k*_, Γ and α, which were not part of the training dataset, we generated coherent scattering intensities using both the GLW and KAN models to assess the accuracy of the KAN-based inversion algorithm. The comparison, presented in Fig. 4 ▸, demonstrates excellent quantitative agreement between the *I*(*Q*) values generated by GLW (colored open symbols) and those produced by KAN (solid curves) across the examined ranges of σ_*k*_, Γ and α. This close correspondence underscores the numerical precision and robustness of the KAN-based regression method.

## 4. Experimental case study of distorted lamellar phases

Having benchmarked the numerical accuracy of the KAN-based inversion algorithm, we now evaluate its feasibility for analyzing real SAS experimental data. We used a least-squares regression analysis (Tung *et al.*, 2024*c* ▸) to match the KAN-generated scattering intensity to the SANS data and obtain the optimized conformation parameters. To this end, a conformational study of a well-known lyotropic system, sodium dioctyl sulfosuccinate (AOT) (Rogers & Winsor, 1969 ▸), was conducted using SANS. AOT powders were dissolved in deuterium oxide (D_2_O) to prepare aqueous solutions with concentrations of 30, 40 and 50% by weight. SANS measurements were performed using the D22 small-angle diffractometer at the Institut Laue–Langevin. The experiment employed two neutron wavelengths, 6 and 11.5 Å, providing 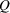-range coverage from 0.001 to 0.5 Å^−1^, where coherent neutron scattering was observed.

**Figure 5:**
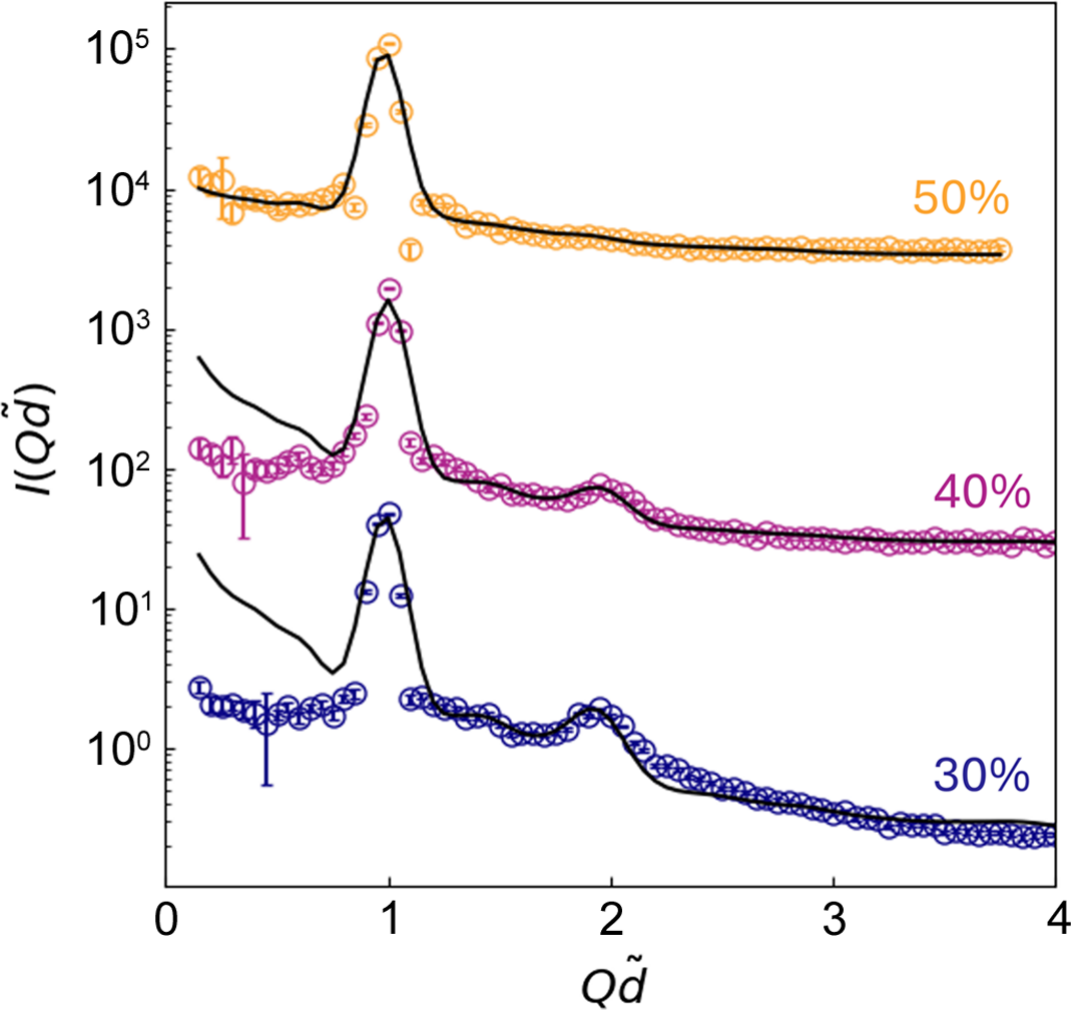
SANS intensity, 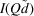, for aqueous solutions of AOT at concentrations of 30, 40 and 50%, presented in dimensionless units with 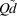 as the key parameter. The initial estimate for the average inter-plane distance, 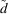, is determined as 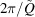, where 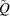 is the position of the first correlation peak in 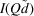. The black curves represent the optimized 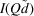 obtained from the KAN model through least-squares regression. Qualitative observations reveal that, as the AOT weight fraction increases, the height of the first correlation peak decreases. Distinct variations in lamellar structural organization are apparent in 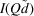, particularly in the height and width of the second correlation peak.

Fig. 5 ▸ displays the SANS intensity, 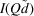, measured for aqueous solutions containing 30, 40 and 50% AOT. The intensity is presented in dimensionless units, with 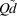 serving as the key parameter. As the AOT weight fraction increases, qualitative trends reveal a reduction in the height of the first correlation peak in 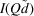. These observations indicate changes in the structural organization of the lamellar phases, consistent with the AOT/water phase diagram (Rogers & Winsor, 1969 ▸). Notably, these structural variations are further reflected in the height and width of the second correlation peak in 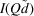.

Quantitative analysis demonstrates that the experimental data (symbols) exhibit strong agreement with the predictions of the KAN model (black curves) in the high-*Q* regime, particularly for 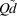 ≳ 1. Minor deviations are noted around the second correlation peak at 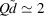. In contrast, significant discrepancies are observed in the low-*Q* regime (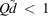). Specifically, the experimental data appear flattened in this region, whereas the KAN model predicts an upturn in 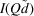, with the intensity of this upturn diminishing as the AOT concentration increases.

Insights into the descriptive framework of the GLW model for distorted lamellar phases can be gained by examining the origins of the observed quantitative discrepancies. First, the instrument resolution can be excluded as the source of these disagreements, as it has been thoroughly addressed in prior work (Huang *et al.*, 2023 ▸).

In the high-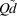 region, we hypothesize that the minor disagreement arises from the ansatz used to quantify the wave vector distribution, as presented in equation (4). Specifically, an alternative formulation of *P*(*k*), characterized by a zero mean but non-zero second and fourth moments (Chen & Choi, 1997 ▸), has been proposed to satisfy the thermodynamic requirement of maximum entropy. This contrasts with our approach, which assumes a non-zero mean alongside second and fourth moments (Berk, 1991 ▸).

Moreover, instead of employing the expression for *P*(θ) given in equation (4) (Fisher, 1953 ▸), a more general formulation involving different bases of Legendre polynomials (Arfken *et al.*, 2012 ▸) could be adopted. This alternative framework would allow for greater flexibility in describing the angular dispersion of **k** by relaxing the degree of freedom along the polar angle direction, thereby providing a model-free description. However, this more general approach introduces additional parameters, including those related to the various polynomials in *P*(*k*) and the coefficients associated with the basis functions of different Legendre polynomial degrees.

The inclusion of these additional parameters would necessitate a significant expansion of the training dataset to effectively map the features of the evolving *I*(*Q*) to the parameters describing the topology of distorted lamellar structures. Consequently, the computational cost of the training process required to establish the KAN for the inversion algorithm would increase exponentially. This phenomenon, often referred to as the ‘curse of dimensionality’ (Bishop, 2006 ▸), highlights the inherent trade-off between model complexity and numerical accuracy in machine-learning approaches when computational resources are limited.

Regarding the disagreement observed in the low-*Q* region, a plausible explanation involves the influence of sample loading on the distributions of grain orientation in AOT solutions. According to findings by Kékicheff *et al.* (1984 ▸), the sample-loading process in lamellar systems notably affects the orientational distribution of grains within the samples, providing a potential explanation for the observed scattering behavior. In the context of a lamellar phase, the sample-loading process may unintentionally induce a non-uniform distribution of orientation among the normal vectors of individual constituent grains, along with potential variations in grain size and inter-grain orientational correlation. This could modify coherent scattering at low values of *Q*. Conversely, in the case of a sponge phase, the sample-loading process has no impact, as the system is inherently isotropic. This argument clarifies why the discrepancy is most evident in the case of a 30% AOT solution, which exhibits the most prominent lamellar order, becomes less pronounced in a 40% AOT solution and virtually vanishes in a 50% AOT solution displaying a pronounced sponge-like topology.

**Figure 6:**
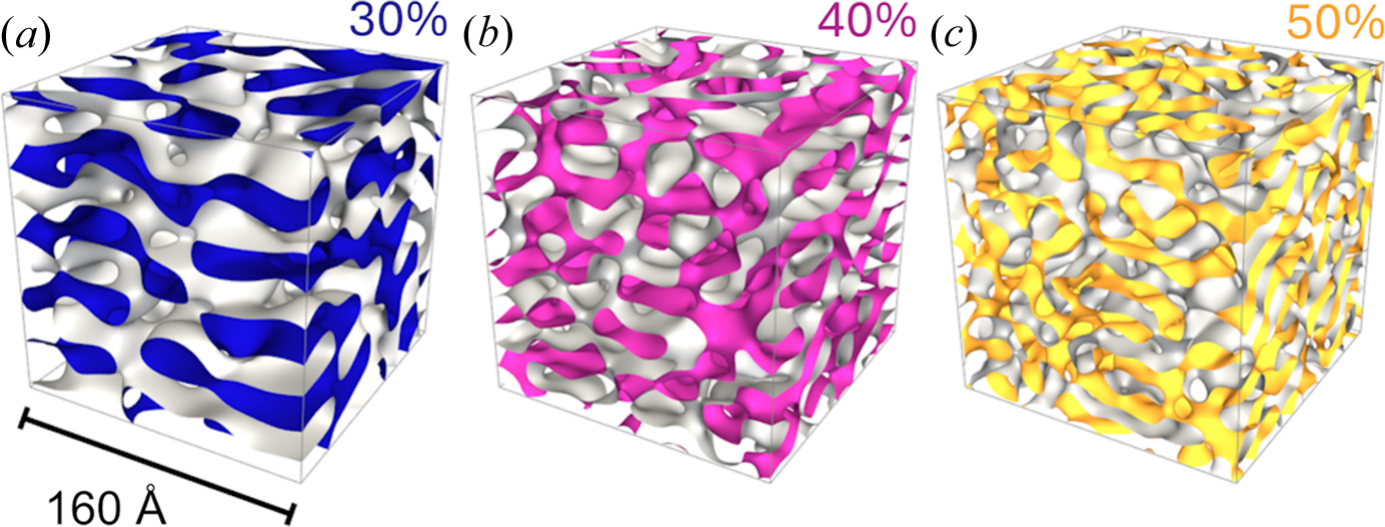
Three-dimensional representation of structures constructed using the extracted σ_*k*_, Γ and α within the GLW framework. The inter-planar spacing is in the range of tens of ångströms. As the AOT weight fraction increases, pathways between adjacent plates develop, enhancing interconnections between layers. This transition leads to a transformation from initially anisotropic, two-dimensional plates to a more isotropic phase at the mesoscopic length scale.

From the extracted values of σ_*k*_, Γ and α, we can construct a three-dimensional representation of the structures within the GLW framework, as illustrated in Fig. 6 ▸. The inter-planar spacing is measured in tens of ångströms. Notably, as the AOT weight fraction increases, pathways between adjacent plates begin to develop, leading to enhanced interconnections between layers. This process gradually transforms the initially anisotropic, two-dimensional plates into a more isotropic phase at the mesoscopic length scale. The detailed topological features of these distorted lamellar phases, including the inter-layer distance, in-plane correlation lengths and local curvatures, can be directly calculated from the real-space renderings shown in Fig. 6 ▸. For further details, readers are referred to Tung *et al.* (2024*c* ▸).

## 5. Identification of defects in distorted lamellar phases

Before introducing the method for defect identification, it is instructive to examine the wave field representation of distorted lamellar phases. To classify topological defects in systems characterized by an order parameter, de Gennes proposed that the dimensionality of a defect must satisfy the following inequality (de Gennes, 1972 ▸; Pieranski, 2019 ▸): 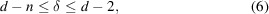
where *d* denotes the spatial dimension of the system, *n* is the number of components of the order parameter and δ represents the dimensionality of the permissible singularities.

In the case of distorted lamellar phases, the spatial dimension is *d* = 3. In the wave field representation, the system has two degrees of freedom corresponding to the real and imaginary parts of the order parameter, giving *n* = 2. Substituting these values into the inequality yields δ = 1. Consequently, the most relevant defect in a distorted lamellar system is a linear discontinuity. Note that the order parameter mentioned here is a function of *r*, which represents the spatial heterogeneity within the system, in contrast to the real-valued, system-level parameter Γ used in equation (4). This topological argument aligns with experimental observations, as TEM studies have demonstrated that topological defects in distorted lamellar phases can manifest as screw dislocations (Kléman *et al.*, 1977 ▸).

In the wave field representation, for a given partial wave, the phase shift ϕ in equation (1) is interpreted as a sinusoidal modulation of mass density along the wave vector direction 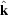. Consequently, differences in the phase shift correspond to the translation of layers. Specifically, along a trajectory encircling a discontinuity – where the real and imaginary components of the partial waves converge to zero amplitude – the phase undergoes a change equal to an integer multiple of 2π, representing the displacement of multiple layers.

To systematically identify defects in a distorted lamellar structure, a three-dimensional wave field *S*(**r**) is analyzed using a grid-based discretization method (Tung *et al.*, 2024*a* ▸). The continuous field *S*(**r**) is discretized onto a cubic grid of size *N* × *N* × *N*, enabling the efficient detection of zeros in the wave field.

**Figure 7:**
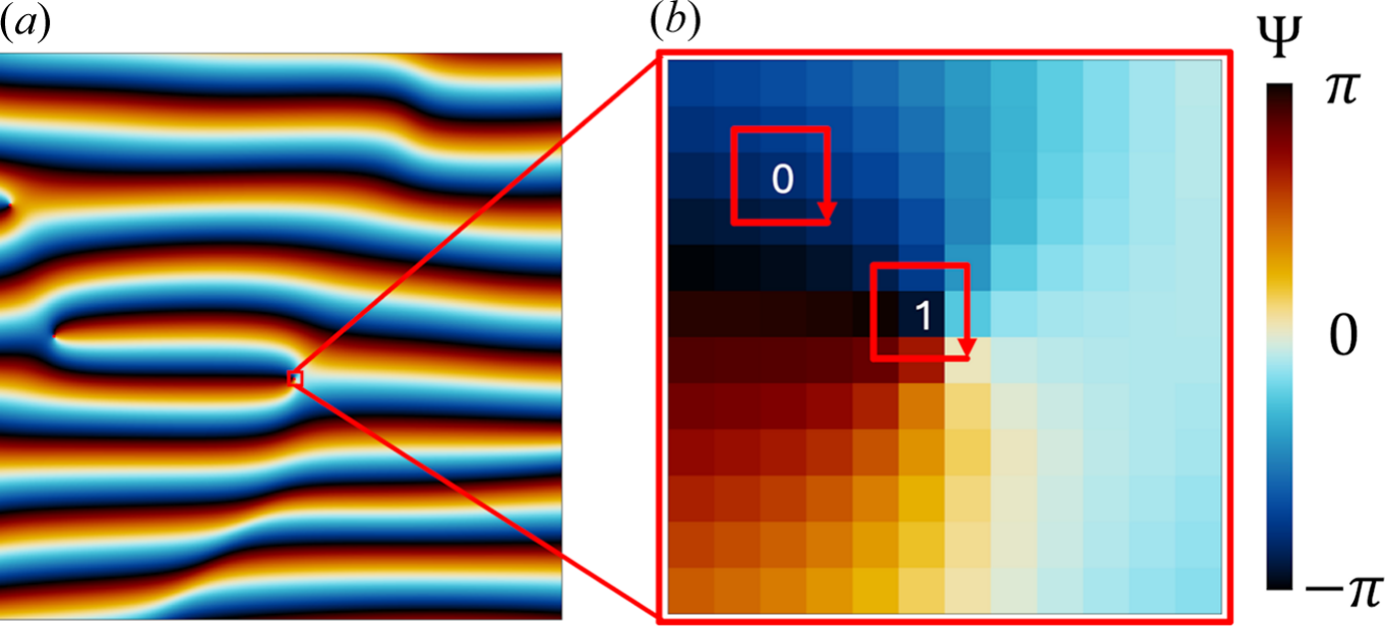
Illustration of topological defect identification in lamellar phases: (*a*) Two-dimensional representation of the phase field Ψ(**r**), derived from the inverted wave field *S*(**r**), visualized using a color gradient to highlight the phase distribution. (*b*) Magnified view of (*a*) showing the circulation path (red squares) used to compute the cumulative phase differences *c*. Pixels where the contour integral of the phase differences are equal to zero are identified, while non-zero values delineate lines of phase discontinuity, emphasizing the defect locations.

The analysis starts with the computation of the argument field Ψ(**r**), which is derived from the argument of the complex wave field *S*(**r**) and is defined as 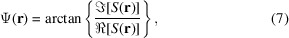
where 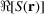 and 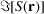 are the real and imaginary components of *S*(**r**), respectively. The computation employs the atan2 function for accurate phase determination (Hochstadt, 1961 ▸). A two-dimensional visualization of the resulting phase field is shown in Fig. 7 ▸(*a*).

To locate lines of phase discontinuity, the discretized argument field Ψ(**r**) is analyzed in two-dimensional slices of the three-dimensional grid. For each pixel within a slice, the cumulative phase difference *c* is calculated along a closed loop formed by the eight neighboring pixels, as illustrated in Fig. 7 ▸(*b*). This cumulative difference is given by
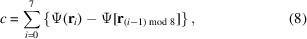
where **r**_*i*_ denotes the position of the *i*th pixel along the loop and Ψ(**r**_*i*_) represents the phase value at that pixel. The modulo operation ensures that the calculation is invariant to the choice of the starting pixel.

This approach enables the systematic identification of phase discontinuities, which correspond to defect lines in the lamellar structure. Figs. 7 ▸(*a*) and 7 ▸(*b*) illustrate the circulation path and its role in detecting these topological defects.

To satisfy the quantization condition of the contour integral of phase differences by 2π, each pixel is labeled as ‘type 0’ if the nearest integer of *c*/2π is zero, and as ‘type 1’ otherwise. This labeling is applied iteratively across all pixels in each slice, continuing through subsequent slices until the entire simulation cell is processed.

To avoid missing lines of phase discontinuity being perpendicular to the evaluation plane, systematic scans are performed along the *x*, *y* and *z* axes. This ensures phase singularities are always encircled by at least one of the loops on the *xy*, *yz* or *zx* planes. The results from these scans are consolidated by the union of the set of defect pixels identified in the three directions.

**Figure 8:**
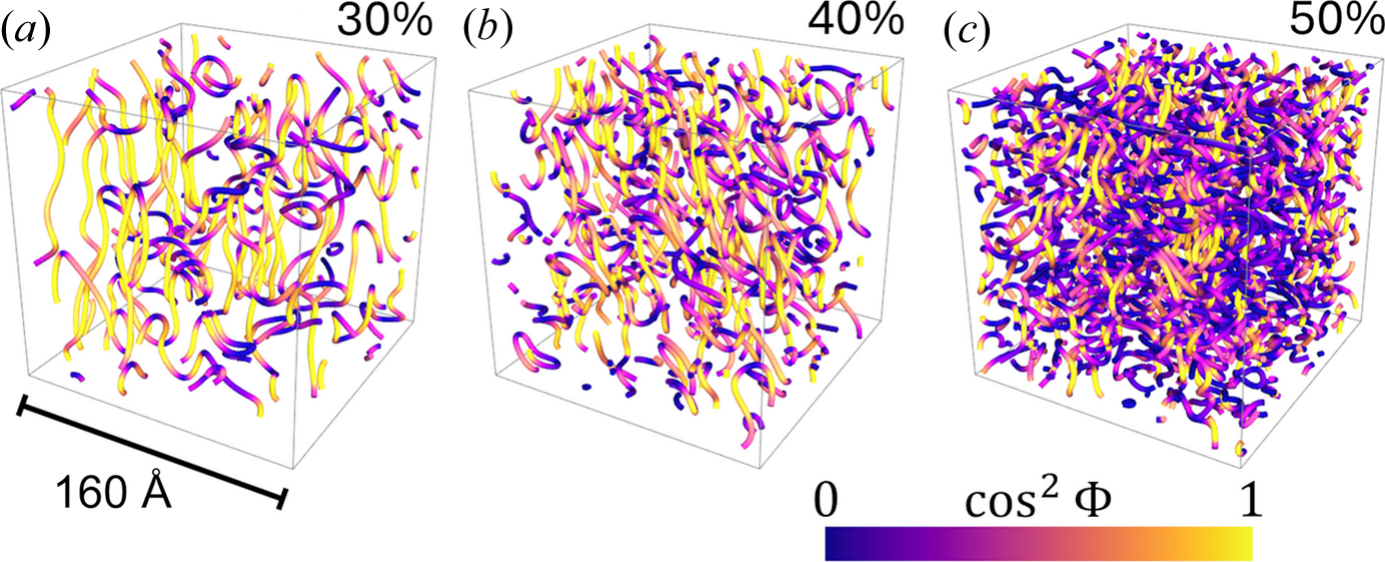
Three-dimensional arrays of lines of linear discontinuity for (*a*) 30%, (*b*) 40% and (*c*) 50% AOT concentrations. The color scheme indicates the direction of the line segments, which represent the topological defects observed in the lamellar phases.

A coarse-graining process is then applied to transform the dispersion of type 1 pixels into a one-dimensional line using a curve-tracing method (Yan, 2001 ▸; Liu *et al.*, 2020 ▸) based on a depth-first search algorithm (Even, 2011 ▸). Details of the technical process can be found in the article by Tung *et al.* (2024*a* ▸). The spatial distribution of linear discontinuities, derived from the analysis of Ψ(**r**) corresponding to 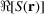 (Fig. 6 ▸), is shown in Fig. 8 ▸. Here, Φ is the angle between the direction of the coarse-grained line and the *z* axis, with cos Φ representing the *z* component of the unit vector.

Two key observations are evident: first, as the AOT concentration increases, the density of line discontinuities rises noticeably; second, the type of linear discontinuity transitions from screw-type dominance at 30% to edge-type dominance at 50%. Further statistical analysis of these topological defects is given by Tung *et al.* (2024*a* ▸).

## 6. Prospects

Two natural extensions of plane wave superposition emerge from this structural study of distorted lamellar phases.

**Figure 9:**
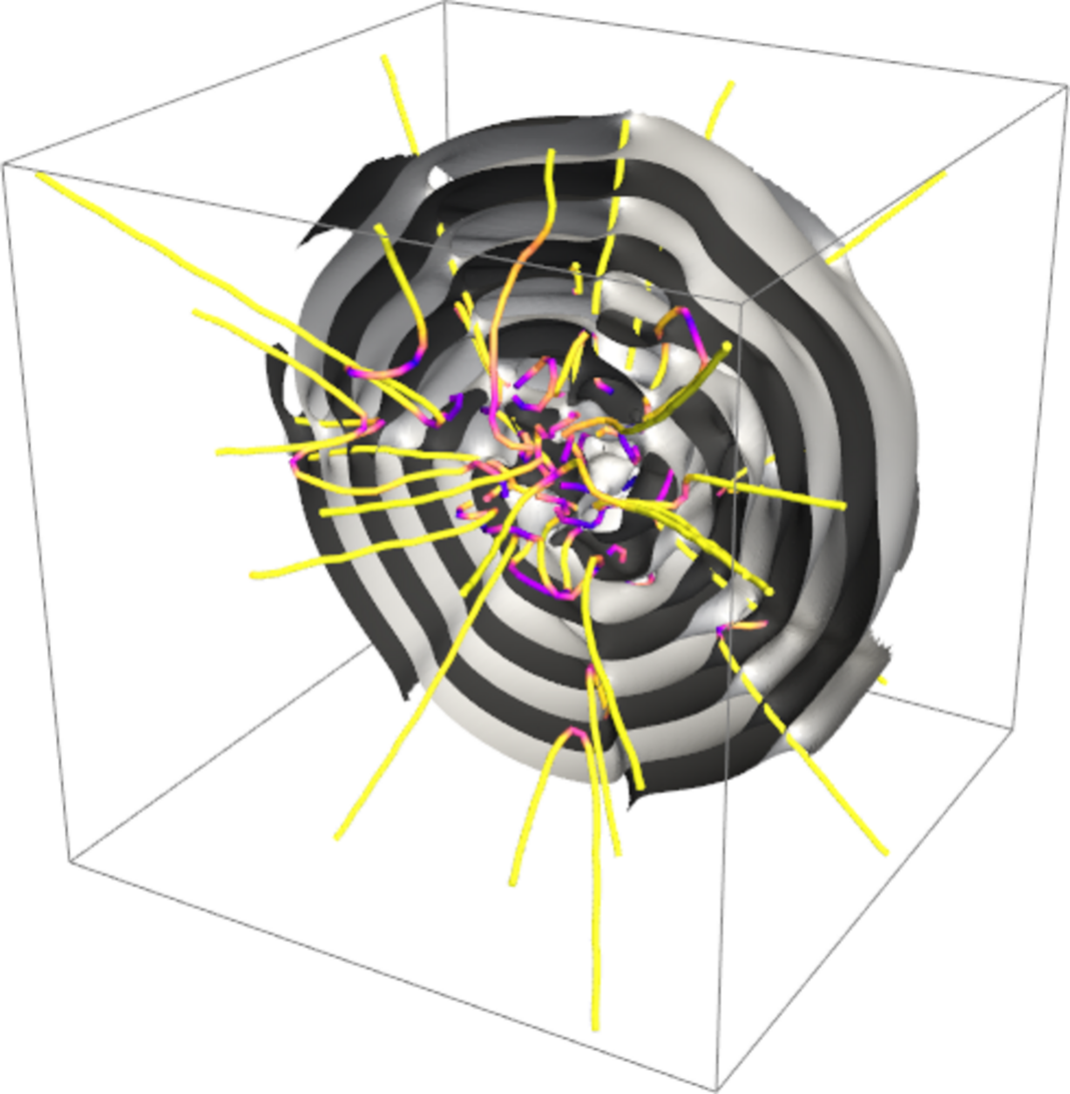
Visualization of a globular onion-like structure generated by the superposition of spherical waves. The structure consists of concentric layers, capturing density fluctuations and mimicking features observed in multilamellar vesicles. The colored lines indicate phase singularities, emphasizing the intricate wave field topology of this configuration. The color gradient illustrates the relationship between the orientation of the line segments and the radial direction.

First, the plane wave basis functions in equation (1) can be replaced by spherical waves as follows:
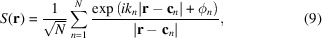
where **c**_*n*_ represents the origins of the individual partial spherical waves. As shown in Fig. 9 ▸, this generalization enables the creation of conformational assemblies resembling globular onion-like structures. Such configurations are valuable for modeling density fluctuations in systems such as multilamellar vesicles, where concentric layers are arranged around an aqueous core.

To generate interference among the partial spherical waves, the origins of individual spherical waves (**c**_*n*_) are designed to exhibit a dispersion around the center of the concentric multilayer structure. Consequently, the aim of the machine-learning process is to map the distribution of **c**_*n*_ from the expressive features of *I*(*Q*), which encode the topological information. This information includes the number of layers, inter-layer spacing, layer curvatures and statistical properties of defects.

This implementation allows for a detailed conformational description of multilayer structures that cannot be adequately captured by conventional core–shell models. By leveraging these capabilities, we advance our understanding of the spatial and topological properties of complex layered systems.

Secondly, understanding the dynamics of topological defects is fundamental for elucidating how imperfections influence the mechanical properties of lamellar systems. By incorporating the temporal oscillation of the wave field into equation (1), the following expression is obtained:
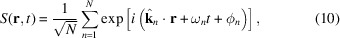
where ω_*n*_ represents the temporal oscillation rate of the *n*th partial wave and *t* is the time. The function *S*(**r**, *t*) describes the dynamics of density fluctuations in distorted lamellar phases at equilibrium.

An experimental approach to determine *S*(**r**, *t*) involves dynamic scattering techniques, such as neutron spin echo (NSE) or X-ray photon correlation spectroscopy (XPCS). In this context, the dispersion relation, which defines the relationship between angular frequency ω and wavevector **k**, plays a crucial role. For defective lamellar phases in AOT solutions, the wavevector probability distribution, *p*(*k*), has been experimentally shown to be well approximated by a Gaussian distribution with a narrow standard deviation (Tung *et al.*, 2024*c* ▸). Consequently, for a given mean wavevector, *k*_0_, in this delta-function-like distribution, the frequency ω(*k*_0_) must follow a specific distribution to enable the lamellar system to initiate ergodic relaxation.

Since the explicit mathematical expression for ω at a given *k*_0_ is currently unknown, a natural starting assumption is that density of state *p*(ω) follows a normal distribution. The goal of the machine-learning process is then to infer the mean and variance of this distribution from experimental features, such as the *Q* dependence of the relaxation time extracted from the intermediate scattering function *F*(*Q*, *t*), which can be experimentally measured.

**Figure 10:**
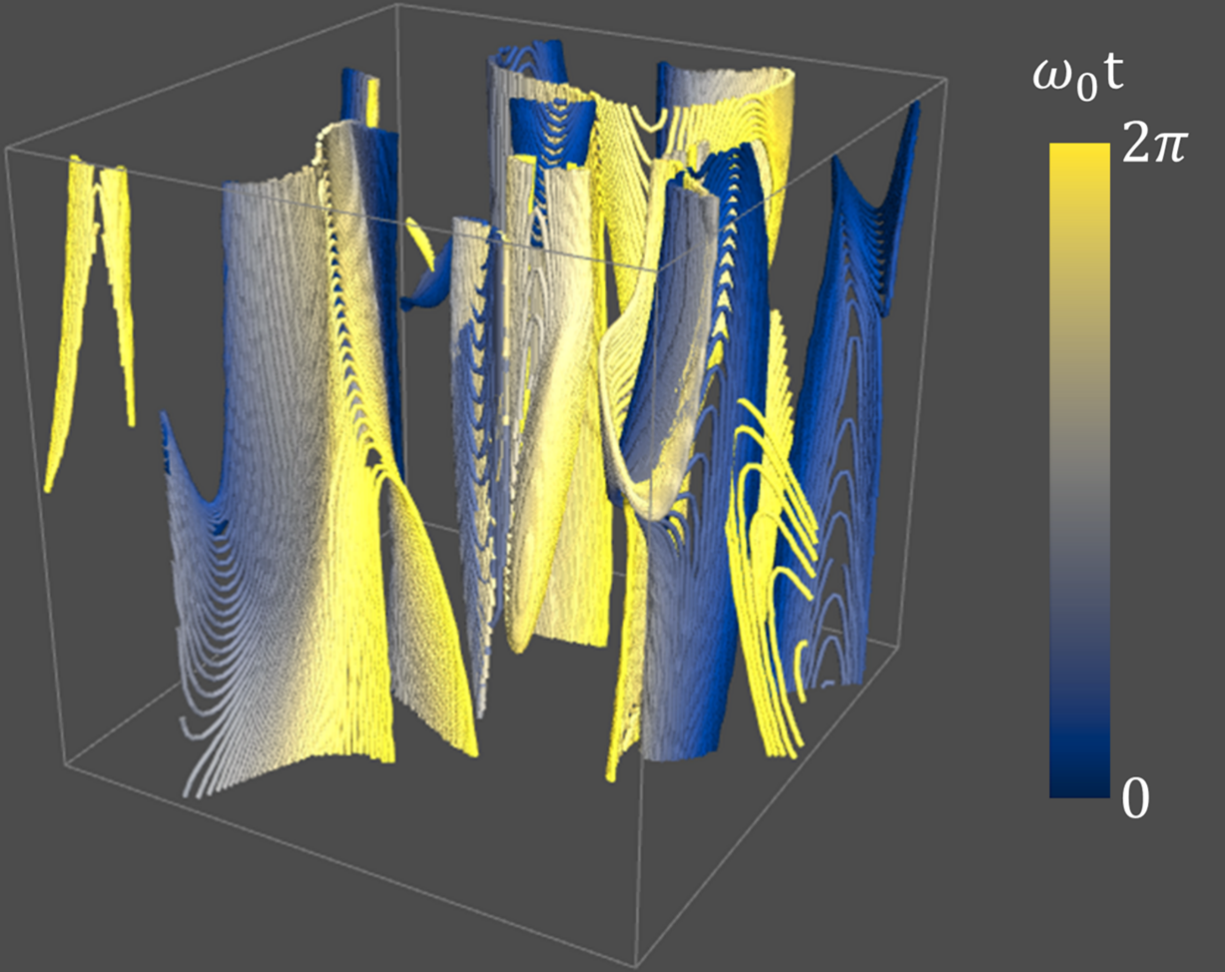
Visualization of the temporal evolution of linear discontinuities in distorted lamellar phases. ω_0_ is the mean of the normal distribution used in this calculation.

Given the mean and variance of the normal distribution derived from the experimental data, the dispersion relation can be determined, which in turn allows for the computation of *S*(**r**, *t*). Through the evaluation of the contour integral, the temporal evolution of linear discontinuities is obtained. An example of such a visualization is provided in Fig. 10 ▸. These trajectories facilitate the calculation of the statistical properties of defect dynamics, thereby opening a new avenue to explore the rheological characteristics of distorted lamellar phases. This approach offers a novel perspective by examining the dynamical heterogeneity of linear discontinuities, providing deeper insights into the complex interplay between defects and material behavior.

## 7. Conclusions

This study demonstrates the synergy between the wave field representation and KAN for analyzing distorted lamellar phases. By employing plane wave superposition to model the anisotropic density fluctuations in lamellar systems, we successfully capture the structural complexity arising from distortions and topological defects. This representation provides a powerful framework for connecting scattering intensity profiles to real-space conformations through the parameters σ_*k*_, Γ and α.

The introduction of KAN complements this approach by offering a robust machine-learning-based inversion method capable of mapping experimental scattering data to structural parameters with high accuracy. Unlike traditional models, KAN’s ability to handle variable experimental *Q* points overcomes significant limitations in regression-based analysis. Validation with SANS data on AOT solutions demonstrated the method’s effectiveness in resolving structural transitions, from ordered lamellar phases to sponge-like morphologies, and in identifying topological defects such as screw and edge dislocations.

Moreover, KAN provides a distinct advantage for SANS users working with lamellar phases by addressing challenges that conventional methods often fail to resolve. The flexibility of KAN in adapting to experimental data with variable *Q* points eliminates the need for potentially error-prone interpolation and extrapolation processes, ensuring more reliable and accurate parameter extraction. For practitioners, this means a significant reduction in analysis time and an increase in the fidelity of the derived structural models, enabling a deeper understanding of complex systems. By facilitating the identification of topological defects and capturing transitions between distinct structural states, KAN opens new possibilities for studying conformational characteristics using scattering and tailoring material properties through controlled defect engineering.

Together, the plane wave superposition framework and KAN establish a versatile toolkit for studying equilibrium and non-equilibrium behavior in lamellar systems. The ability to integrate dynamic scattering techniques, such as NSE or XPCS, with this methodology opens new avenues for understanding defect dynamics and their impact on material properties.

This work advances the field of soft matter physics by bridging theoretical wave field modeling and machine-learning innovations. Future extensions could refine the wave vector distributions or adopt generalized statistical descriptors to broaden the applicability of this framework to other mesostructured materials.

## References

[bb1] Allain, M. (1986). *Europhys. Lett.* **2**, 597–602.

[bb2] Allain, M. & Kléman, M. (1985). *J. Phys. Fr.* **46**, 225–234.

[bb3] Allain, M. & Kléman, M. (1987). *J. Phys. Fr.* **48**, 1799–1807.

[bb4] Angelov, B., Angelova, A., Vainio, U., Garamus, V. M., Lesieur, S., Willumeit, R. & Couvreur, P. (2009). *Langmuir*, **25**, 3734–3742. 10.1021/la804225j 19708151

[bb5] Arfken, G. B., Weber, H. J. & Harris, F. E. (2012). *Mathematical methods for physicists: a comprehensive guide*. Academic Press.

[bb6] Arnol’d, V. I. (1956). *Dokl. Akad. Nauk SSSR*, **108**, 179–182.

[bb7] Baciu, M., Holmes, M. C. & Leaver, M. S. (2007). *J. Phys. Chem. B*, **111**, 909–917. 10.1021/jp066595n 17249835

[bb8] Bartels, R. H., Beatty, J. C. & Barsky, B. A. (1987). *An introduction to splines for use in computer graphics and geometric modeling*. Morgan Kaufmann Publishers.

[bb9] Berk, N. F. (1987). *Phys. Rev. Lett.* **58**, 2718–2721. 10.1103/PhysRevLett.58.2718 10034827

[bb10] Berk, N. F. (1991). *Phys. Rev. A*, **44**, 5069–5079. 10.1103/physreva.44.5069 9906559

[bb11] Bishop, C. M. (2006). *Pattern recognition and machine learning*. Springer.

[bb12] Blanc, C., Meyer, C. S., Asnacios, S., Kléman, M., Lelidis, I. & Martin, J.-L. (2005). *Philos. Mag. Lett.* **85**, 641–648.

[bb13] Boden, N., Clements, J., Jolley, K. W., Parker, D. & Smith, M. H. (1990). *J. Chem. Phys.* **93**, 9096–9105.

[bb14] Boden, N., Corne, S. A., Holmes, M. C., Jackson, P. H., Parker, D. & Jolley, K. W. (1986). *J. Phys. Fr.* **47**, 2135–2144.

[bb15] Boden, N., Corne, S. A. & Jolley, K. W. (1981). *Mol. Cryst. Liq. Cryst.* **67**, 277–282.

[bb16] Boden, N. & Jolley, K. W. (1992). *Phys. Rev. A*, **45**, 8751–8758. 10.1103/physreva.45.8751 9906973

[bb17] Bouglet, G. & Ligoure, C. (1999). *Eur. Phys. J. B*, **9**, 137–147.

[bb18] Bourdon, L., Sommeria, J. & Kléman, M. (1982). *J. Phys. Fr.* **43**, 77–96.

[bb19] Callaghan, P. T. & Soderman, O. (1983). *J. Phys. Chem.* **87**, 1737–1744.

[bb20] Castelletto, V., Fisher, J., Hamley, I. W. & Yang, Z. (2002). *Colloids Surf. A Physicochem. Eng. Asp.* **211**, 9–18.

[bb21] Castro-Roman, F., Porcar, L., Porte, G. & Ligoure, C. (2005). *Eur. Phys. J. E*, **18**, 259–272. 10.1140/epje/e2005-00029-6 16231078

[bb22] Chang, M.-C., Tung, C.-H., Chang, S.-Y., Carrillo, J.-M., Wang, Y., Sumpter, B. G., Huang, G.-R., Do, C. & Chen, W.-R. (2022). *Commun. Phys.* **5**, 1–8.

[bb23] Chen, S.-H. & Choi, S.-M. (1997). *J. Appl. Cryst.* **30**, 755–760.

[bb24] Chidichimo, G., Coppola, L., La Mesa, C., Ranieri, G. A. & Saupe, A. (1988). *Chem. Phys. Lett.* **145**, 85–89.

[bb25] Chidichimo, G., La Mesa, C., Ranieri, G. A. & Terenzi, M. (1987). *Mol. Cryst. Liq. Cryst.* **150b**, 221–236.

[bb26] Coppola, L., Gianferri, R., Nicotera, I. & Oliviero, C. (2003). *Mol. Cryst. Liq. Cryst.* **398**, 157–167.

[bb27] Coppola, L., Muzzalupo, R., Ranieri, G. A. & Terenzi, M. (1995). *Langmuir*, **11**, 1116–1121.

[bb28] Costello, M. J., Meiboom, S. & Sammon, M. (1984). *Phys. Rev. A*, **29**, 2957–2959.

[bb29] Davis, J. H. (1983). *Biochim. Biophys. Acta*, **737**, 117–171. 10.1016/0304-4157(83)90015-1 6337629

[bb30] Dhez, O., König, S., Roux, D., Nallet, F. & Diat, O. (2000). *Eur. Phys. J. E*, **3**, 377–388.

[bb31] Ding, L., Chen, Y. & Do, C. (2024*a*). *arXiv*, 2412.07926.

[bb32] Ding, L., Tung, C.-H., Cao, Z., Ye, Z., Gu, X., Xia, Y., Chen, W.-R. & Do, C. (2024*b*). *arXiv*, 2411.00134.

[bb33] Ding, L., Tung, C.-H., Sumpter, B. G., Chen, W.-R. & Do, C. (2024*c*). *arXiv*, 2410.05574.

[bb34] Doe, C., Jang, H.-S., Kline, S. R. & Choi, S.-M. (2009). *Macromolecules*, **42**, 2645–2650.

[bb35] Eriksson, S., Lasič, S., Nilsson, M., Westin, C.-F. & Topgaard, D. (2015). *J. Chem. Phys.* **142**, 104201. 10.1063/1.4913502 PMC4359170 25770532

[bb36] Even, S. (2011). *Graph algorithms*. Cambridge University Press.

[bb37] Fairhurst, C. E., Holmes, M. C. & Leaver, M. S. (1997). *Langmuir*, **13**, 4964–4975.

[bb38] Fisher, R. A. (1953). *Proc. R. Soc. London Ser. A*, **217**, 295–305.

[bb39] Funari, S. S., Holmes, M. C. & Tiddy, G. J. T. (1992). *J. Phys. Chem.* **96**, 11029–11038.

[bb40] Funari, S. S., Holmes, M. C. & Tiddy, G. J. T. (1994). *J. Phys. Chem.* **98**, 3015–3023.

[bb41] Gennes, P.-G. de (1972). *C. R. Hebd. Séances Acad. Sci. Ser. B*, **275**, 319–321.

[bb42] Hamley, I. W. (2022). *Soft Matter*, **18**, 711–721. 10.1039/d1sm01758f 35014650

[bb43] Hendrikx, Y., Charvolin, J., Kékicheff, P. & Roth, M. (1987). *Liq. Cryst.* **2**, 677–687.

[bb44] Hendrikx, Y., Charvolin, J. & Rawiso, M. (1984). *J. Colloid Interface Sci.* **100**, 597–600.

[bb45] Hochstadt, H. (1961). *Special functions of mathematical physics*. Holt, Rinehart and Winston.

[bb46] Holmes, M. C. & Charvolin, J. (1984). *J. Phys. Chem.* **88**, 810–818.

[bb47] Holmes, M. C., Charvolin, J. & Reynolds, D. J. (1988). *Liq. Cryst.* **3**, 1147–1155.

[bb48] Holmes, M. C., Reynolds, D. J. & Boden, N. (1987). *J. Phys. Chem.* **91**, 5257–5262.

[bb49] Holmes, M. C., Smith, A. M. & Leaver, M. S. (1993). *J. Phys. II Fr.* **3**, 1357–1370.

[bb50] Huang, G.-R., Tung, C.-H., Chen, M.-Z., Porcar, L., Shinohara, Y., Wildgruber, C. U., Do, C. & Chen, W.-R. (2023). *J. Appl. Cryst.* **56**, 1537–1543. 10.1107/S1600576725004212 PMC12321025 40765981

[bb51] Hubbard, P. L., McGrath, K. M. & Callaghan, P. T. (2005). *Langmuir*, **21**, 4340–4346. 10.1021/la0470378 16032845

[bb52] Jóhannesson, H., Furó, I. & Halle, B. (1996). *Phys. Rev. E*, **53**, 4904–4917. 10.1103/physreve.53.4904 9964819

[bb56] Kékicheff, P. (1989). *J. Colloid Interface Sci.* **131**, 133–152.

[bb57] Kékicheff, P. & Cabane, B. (1988). *Acta Cryst.* B**44**, 395–406.

[bb58] Kékicheff, P., Cabane, B. & Rawiso, M. (1984). *J. Phys. Lett.* **45**, 813–821.

[bb59] Kékicheff, P. & Tiddy, G. J. T. (1989). *J. Phys. Chem.* **93**, 2520–2526.

[bb53] Kléman, M. (1989). *Rep. Prog. Phys.* **52**, 555–654.

[bb54] Kléman, M., Williams, C. E., Costello, M. J. & Gulik-krzywicki, T. (1977). *Philos. Mag.* **35**, 33–56.

[bb55] Kolmogorov, A. N. (1957). *Dokl. Akad. Nauk SSSR*, **114**, 679–681.

[bb60] Leaver, M. S. & Holmes, M. C. (1993). *J. Phys. II Fr.* **3**, 105–120.

[bb61] Lemmich, J., Mortensen, K., Ipsen, J. H., Hønger, T., Bauer, R. & Mouritsen, O. G. (1996). *Phys. Rev. E*, **53**, 5169–5180. 10.1103/physreve.53.5169 9964849

[bb62] Liu, D., Xiong, C. & Liu, X. (2020). *IEEE Trans. Vis. Comput. Graph.* **27**, 3794–3807. 10.1109/TVCG.2020.2981460 32191891

[bb63] Liu, Z., Wang, Y., Vaidya, S., Ruehle, F., Halverson, J., Soljačić, M., Hou, T. Y. & Tegmark, M. (2024). *arXiv*, 2404.19756.

[bb64] Meklesh, V. & Kékicheff, P. (2021). *J. Colloid Interface Sci.* **582**, 1158–1178. 10.1016/j.jcis.2020.08.034 32949921

[bb65] Meyer, R. B., Stebler, B. & Lagerwall, S. T. (1978). *Phys. Rev. Lett.* **41**, 1393–1395.

[bb66] Mihailescu, M., Monkenbusch, M., Allgaier, J., Frielinghaus, H., Richter, D., Jakobs, B. & Sottmann, T. (2002). *Phys. Rev. E*, **66**, 041504. 10.1103/PhysRevE.66.041504 12443209

[bb67] Minewaki, K., Kato, T., Yoshida, H., Imai, M. & Ito, K. (2001). *Langmuir*, **17**, 1864–1871.

[bb68] Moreau, P., Navailles, L., Giermanska-Kahn, J., Mondain-Monval, O., Nallet, F. & Roux, D. (2006). *Europhys. Lett.* **73**, 49–54.

[bb69] Nallet, F., Laversanne, R. & Roux, D. (1993). *J. Phys. II Fr.* **3**, 487–502.

[bb70] Orädd, G., Gustafsson, J. & Almgren, M. (2001). *Langmuir*, **17**, 3227–3234.

[bb71] Pabst, G., Koschuch, R., Pozo-Navas, B., Rappolt, M., Lohner, K. & Laggner, P. (2003). *J. Appl. Cryst.* **36**, 1378–1388.

[bb72] Pabst, G., Rappolt, M., Amenitsch, H. & Laggner, P. (2000). *Phys. Rev. E*, **62**, 4000–4009. 10.1103/physreve.62.4000 11088921

[bb73] Paz, L., Di Meglio, J. M., Dvolaitzky, M., Ober, R. & Taupin, C. (1984). *J. Phys. Chem.* **88**, 3415–3418.

[bb74] Photinos, P. & Saupe, A. (1991). *Phys. Rev. A*, **43**, 2890–2896. 10.1103/physreva.43.2890 9905354

[bb75] Photinos, P. J. & Saupe, A. (1986). *J. Chem. Phys.* **84**, 517–521.

[bb76] Photinos, P. J., Yu, L. J. & Saupe, A. (1981). *Mol. Cryst. Liq. Cryst.* **67**, 277–281.

[bb77] Pieranski, P. (2019). *C. R. Phys.* **20**, 756–769.

[bb78] Porte, G. (2002). *Neutron, X-ray and light: scattering methods applied to soft condensed matter*, edited by Th. Zemb & P. Lindner, pp. 299–315. North Holland.

[bb79] Prévost, S., Gradzielski, M. & Zemb, Th. (2017). *Adv. Colloid Interface Sci.* **247**, 374–396. 10.1016/j.cis.2017.07.022 28780230

[bb80] Quest, P., Fontell, K. & Halle, B. (1994). *Liq. Cryst.* **16**, 235–256.

[bb81] Rogers, J. & Winsor, P. A. (1969). *J. Colloid Interface Sci.* **30**, 247–257.

[bb82] Spinozzi, F. & Amaral, L. Q. (2016). *Langmuir*, **32**, 13556–13565. 10.1021/acs.langmuir.6b04124 27993017

[bb83] Spinozzi, F., Paccamiccio, L., Mariani, P. & Amaral, L. Q. (2010). *Langmuir*, **26**, 6484–6493. 10.1021/la9039623 20180590

[bb84] Strey, R., Jahn, W., Porte, G. & Bassereau, P. (1990). *Langmuir*, **6**, 1635–1639.

[bb85] Tung, C., Chang, S., Chang, M., Carrillo, J., Sumpter, B. G., Do, C. & Chen, W.. (2023). *Carbon Trends*, **10**, 100252.

[bb86] Tung, C. H., Chang, S. Y., Chen, H. L., Wang, Y., Hong, K., Carrillo, J. M., Sumpter, B. G., Shinohara, Y., Do, C. & Chen, W. R. (2022). *J. Chem. Phys.* **156**, 131101. 10.1063/5.0086311 35395880

[bb87] Tung, C., Chen, H., Huang, G., Porcar, L., Impéror, M., Carrillo, J. Y., Wang, Y., Sumpter, B. G., Shinohara, Y., Taylor, J., Do, C. & Chen, W. (2024*a*). *Macromolecules*, **57**, 6979–6989.

[bb88] Tung, C.-H., Chen, M.-Z., Chen, H.-L., Huang, G.-R., Porcar, L., Chang, M.-C., Carrillo, J.-M., Wang, Y., Sumpter, B. G., Shinohara, Y., Do, C. & Chen, W.-R. (2024*b*). *J. Appl. Cryst.* **57**, 1047–1058.

[bb89] Tung, C. H., Hsiao, Y. J., Chen, H. L., Huang, G. R., Porcar, L., Chang, M. C., Carrillo, J. M., Wang, Y., Sumpter, B. G., Shinohara, Y., Taylor, J., Do, C. & Chen, W. R. (2024*c*). *J. Colloid Interface Sci.* **659**, 739–750. 10.1016/j.jcis.2024.01.003 38211491

[bb90] Turing, A. M. (1952). *Philos. Trans. R. Soc. London B*, **237**, 37–72.

[bb91] Ukleja, P., Chidichimo, G. & Photinos, P. (1991). *Liq. Cryst.* **9**, 359–367.

[bb92] Vonk, C. G. (1978). *J. Appl. Cryst.* **11**, 541–546.

[bb93] Yamashita, I., Kawabata, Y., Kato, T., Hato, M. & Minamikawa, H. (2004). *Colloids Surf. A Physicochem. Eng. Asp.* **250**, 485–490.

[bb94] Yan, H. (2001). *IEEE Trans. Syst. Man Cybern.* **31**, 768–780. 10.1109/3477.956038 18244841

[bb95] Zemb, Th. (2002). *Neutron, X-ray and light: scattering methods applied to soft condensed matter*, edited by Th. Zemb & P. Lindner, pp. 317–350. North Holland.

[bb96] Zhang, C., Gao, M., Diorio, N., Weissflog, W., Baumeister, U., Sprunt, S., Gleeson, J. T. & Jákli, A. (2012). *Phys. Rev. Lett.* **109**, 107802. 10.1103/PhysRevLett.109.107802 23005329

[bb97] Zhang, R., Suter, R. M. & Nagle, J. F. (1994). *Phys. Rev. E*, **50**, 5047–5060. 10.1103/physreve.50.5047 9962590

